# Severe pneumonia due to *Cupriavidus gilardii* in a critically ill patient: a case report highlighting therapeutic dilemmas and the imperative for standardized antimicrobial guidance

**DOI:** 10.3389/fmed.2025.1732674

**Published:** 2026-02-19

**Authors:** Gengchen Huang, Shuxin Li, Yitong Zhang, Binbin Wang, Lihuan Zhang, Yutao Ma, Zihan Gao, Wei Wei

**Affiliations:** 1 Department of Urology II, The First Hospital of Jilin University, Changchun, China; 2 Department of Neurology, The First Affiliated Hospital of Jinzhou Medical University, Jinzhou, China

**Keywords:** antimicrobial stewardship, *C. gilardii*, case report, drug resistance, microbial identification, severe pneumonia

## Abstract

*Cupriavidus gilardii* is a rare environmental Gram-negative bacillus that has increasingly been recognized as an opportunistic pathogen in recent years. Clinical management of infections caused by this microorganism remains challenging due to difficulties in identification and the lack of standardized guidelines for antimicrobial susceptibility testing (AST). This article reports the case of a 75-year-old male with severe pneumonia and multiple comorbidities. *Cupriavidus gilardii* meeting the quality standards was identified in both bronchoalveolar lavage fluid (BALF) and sputum cultures. However, conventional AST could not be performed on this rare isolation. In the absence of susceptibility data, initial empirical therapy consisted of meropenem combined with amphotericin B cholesteryl sulfate complex. Based on literature review and microbiological findings, the regimen was later adjusted to cefoperazone–sulbactam (Sulperazon) combined with minocycline. Targeted treatment led to marked improvement in inflammatory markers and chest imaging. However, therapy was discontinued prematurely at the family’s request due to financial constraints, and the patient was transferred to a local hospital before full recovery could be achieved. This case demonstrates that *C. gilardii* can cause severe, life-threatening pneumonia in critically ill patients with multiple comorbidities, challenging its perception as a low-virulence opportunist. The absence of standardized antimicrobial susceptibility testing for this pathogen necessitated a literature-guided, multidisciplinary therapeutic approach, which ultimately led to clinical improvement. Our experience underscores the urgency of establishing standardized testing protocols and fostering multidisciplinary collaboration to guide the management of emerging, multidrug-resistant environmental pathogens like *C. gilardii*.

## Introduction

*Cupriavidus gilardii* is an aerobic, non-fermentative, Gram-negative bacillus belonging to the Burkholderiaceae family. It is characterized by its inability to ferment glucose, while typically testing positive for oxidase and catalase. These conventional biochemical profiles, however, offer limited discriminatory power and can easily lead to misidentification as other non-fermentative rods, such as *Cupriavidus pauculus* or *Cupriavidus nantongensis* species, underscoring the diagnostic challenge at the genus and species level (1). First identified by Coenye et al. in 1999, this microorganism is commonly found in diverse environmental sources, including heavy-metal-contaminated plants and soil (2–5). Phenotypically, it can grow on MacConkey agar and is often motile. Although it has been isolated from human clinical specimens—including cerebrospinal fluid, bone marrow, wounds, boils, and the respiratory tract—its clinical significance remains poorly understood (6, 7). To date, *C. gilardii* has been primarily associated with opportunistic infections in immunocompromised individuals, and AST often reveals resistance or intermediate susceptibility to various antibiotics (8, 9). Genomic studies have identified two novel strains, NOV2-1 and OV2-1, that possess a secondary chromosome or mega plasmid and exhibit thermotolerance up to 48 °C, a potential hallmark of *C. gilardii* (10). In clinical diagnostics, whole-genome sequencing (WGS) has emerged as a practical tool for accurate species identification (1). However, the management of *C. gilardii* infections remains challenging due to the absence of standardized AST guidelines. Herein, we present a case of severe pneumonia secondary to aspiration, with subsequent isolation of *C. gilardii*. This report aims to illustrate the diagnostic and therapeutic challenges posed by this pathogen, discuss the clinical decision-making process based on limited literature, and contribute to the growing body of evidence on rare pathogens, thereby underscoring the imperative for multidisciplinary management and early suspicion in vulnerable hosts.

## Case description

**Figure 1:**
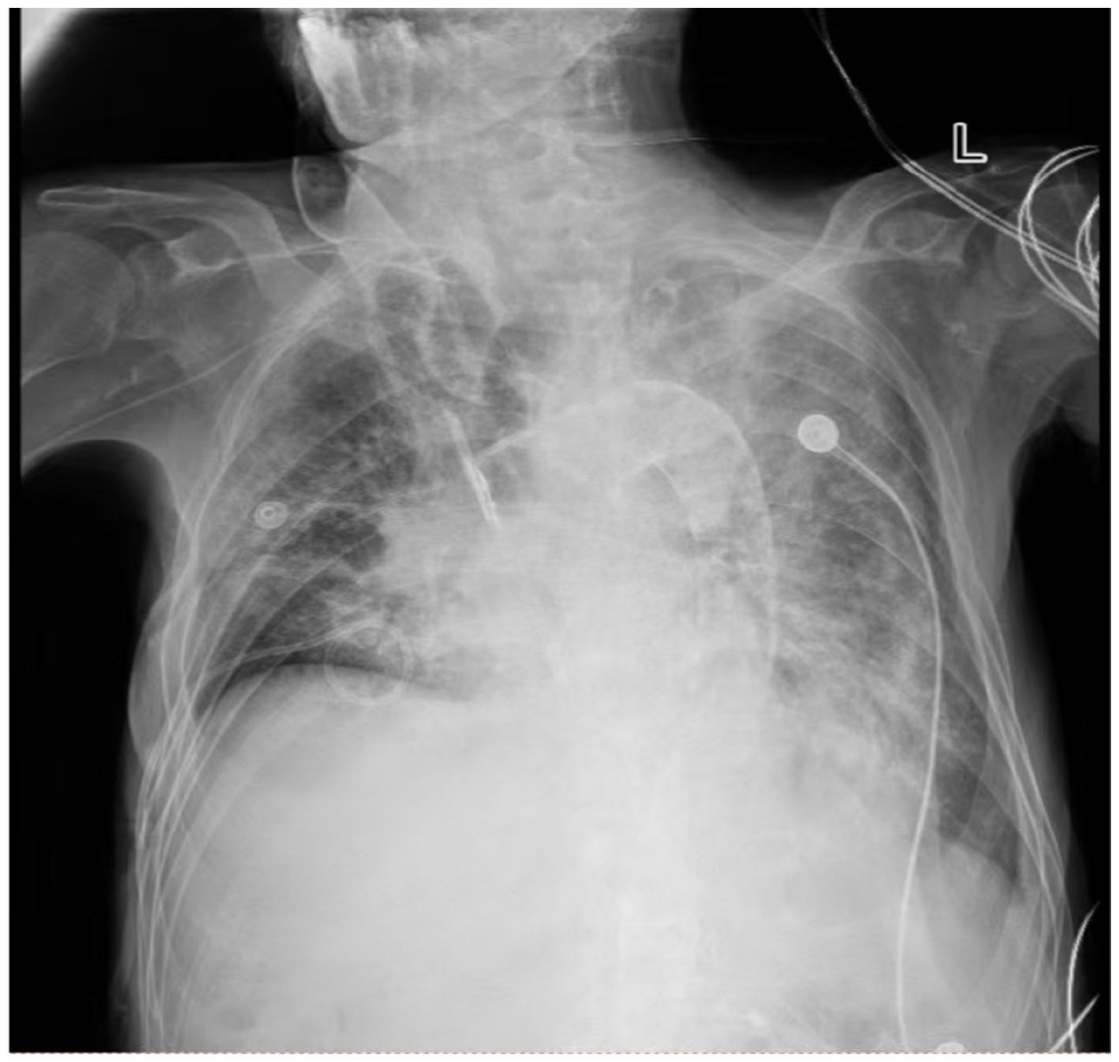
Bedside chest radiograph (DR) of the patient. The image demonstrates increased lung markings and patchy areas of consolidation in both lung fields.

**Figure 2:**
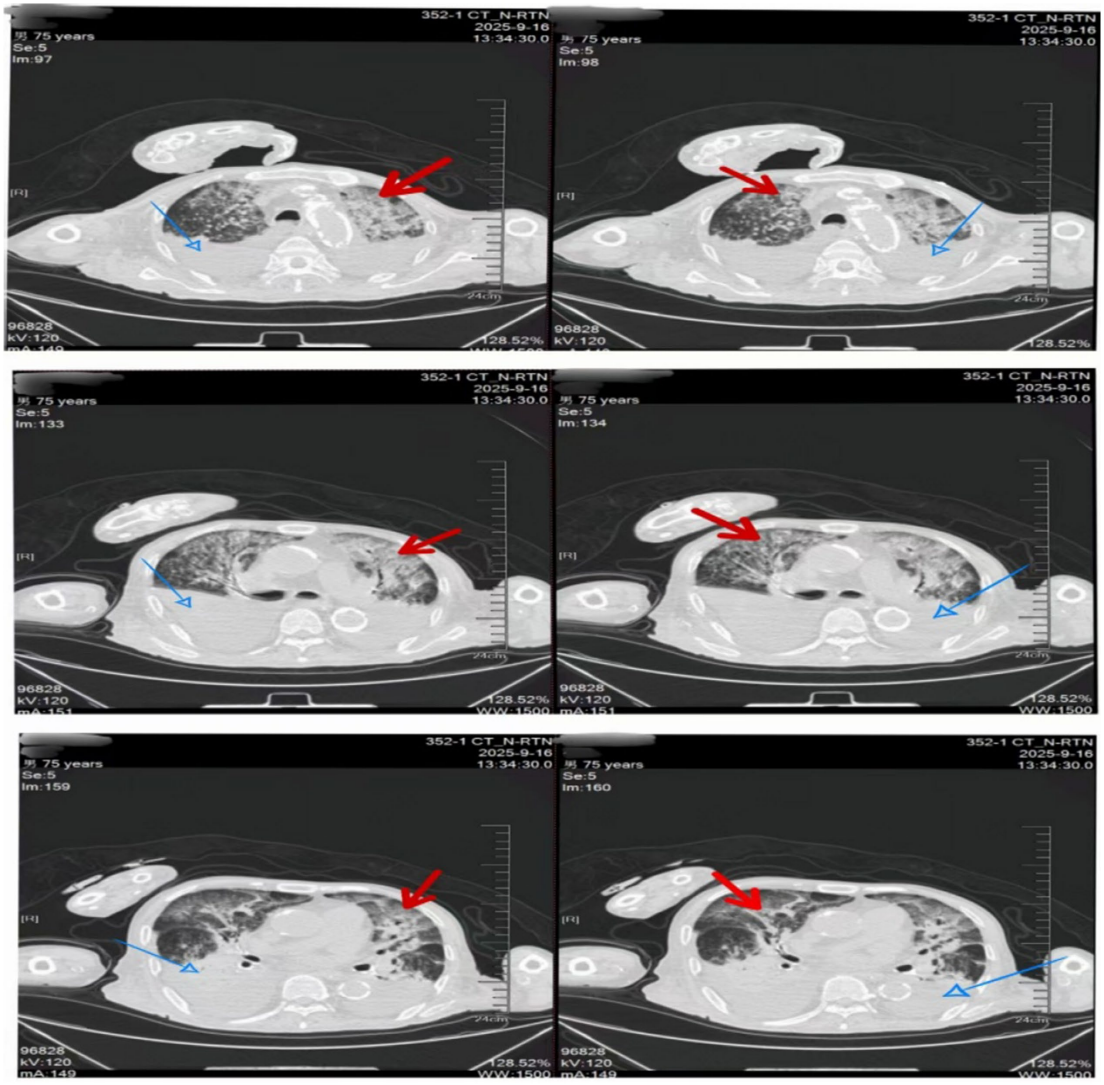
Multidetector computed tomography (MDCT) of the patient’s chest. Blue arrows indicate reduced lung volume and atelectasis in the lower lobes of both lungs. Red arrows demonstrate scattered high-density opacities bilaterally, consistent with pulmonary pneumonia.

A 75-year-old male was admitted to the First Hospital of Jilin University on September 16, 2025, due to progressive dyspnea for 1 week. His symptoms began 1 week prior with choking and dyspnea following nasogastric tube feeding initiated at a local hospital for anorexia. This progressed to hypoxemia. During his illness, the patient experienced impaired consciousness, intermittent cough, and productive sputum, with anuria. A chest CT from the referring hospital indicated bilateral pneumonia, and bronchoscopy revealed gastric content aspiration, leading to a diagnosis of aspiration pneumonia and subsequent transfer to our hospital for further management. The patient’s past medical history was significant for chronic renal failure on maintenance hemodialysis for 14 years, hypertension, and status post-bilateral lower limb amputation. Over the preceding 3 weeks, he had experienced markedly reduced oral intake, was bedridden with impaired consciousness, and was in a state of severe malnutrition (serum albumin <27 g/L). On admission, physical examination revealed: temperature 36.2 °C, respiratory rate 35 breaths/min, blood pressure 150/85 mmHg, and oxygen saturation 85% (on a reservoir mask at 10 L/min). The patient was lethargic. Pulmonary examination demonstrated dullness to percussion and diffuse wet rales bilaterally upon auscultation. Laboratory tests were consistent with severe infection: white blood cell count 21.58 × 10^9^/L, neutrophil percentage 93%, high-sensitivity C-reactive protein 163.97 mg/L, procalcitonin 7.2 ng/mL, and interleukin-6 (IL-6) 104.02 pg/mL. Fungal (1,3)-*β*-D-glucan was elevated to 109.35 pg/mL (positive). Testing for 13 common respiratory pathogens and SARS-CoV-2 nucleic acid was negative. Pathogen DNA detection from sputum was positive only for *Stenotrophomonas maltophilia*. A bedside chest X-ray confirmed bilateral pneumonia (Figure 1). Multidetector computed tomography (MDCT) of the chest revealed retained secretions in the trachea and left main bronchus, along with diffuse bilateral pulmonary infiltrates (Figure 2).

### Microbiological identification and laboratory criteria

Both BALF and sputum samples were collected aseptically and processed immediately for routine bacterial culture. The sputum specimen met quality standards for lower respiratory tract samples (white blood cells >25 per low-power field and epithelial cells <10 per low-power field). Bacterial identification was performed on the isolated pure colonies using matrix-assisted laser desorption/ionization time-of-flight mass spectrometry (MALDI-TOF MS). This identification was performed by the hospital’s clinical microbiology laboratory in accordance with its standardized operating procedures. *Cupriavidus gilardii* was identified in both BALF and sputum cultures with a high confidence score (≥2.0), and the identification results of the two independent samples were consistent. Since *C. gilardii* is a rare pathogen and there is a lack of standardized drug susceptibility test methods and interpretation criteria, the routine testing items of the clinical microbiology laboratory in our hospital cannot conduct effective antimicrobial susceptibility tests on it. The concurrent detection of *C. gilardii* from two independent respiratory specimens, in the context of severe clinical and radiological signs of pneumonia, supports its role as a probable pathogen rather than mere colonization or contamination.

### Clinical course and management

**Figure 3:**
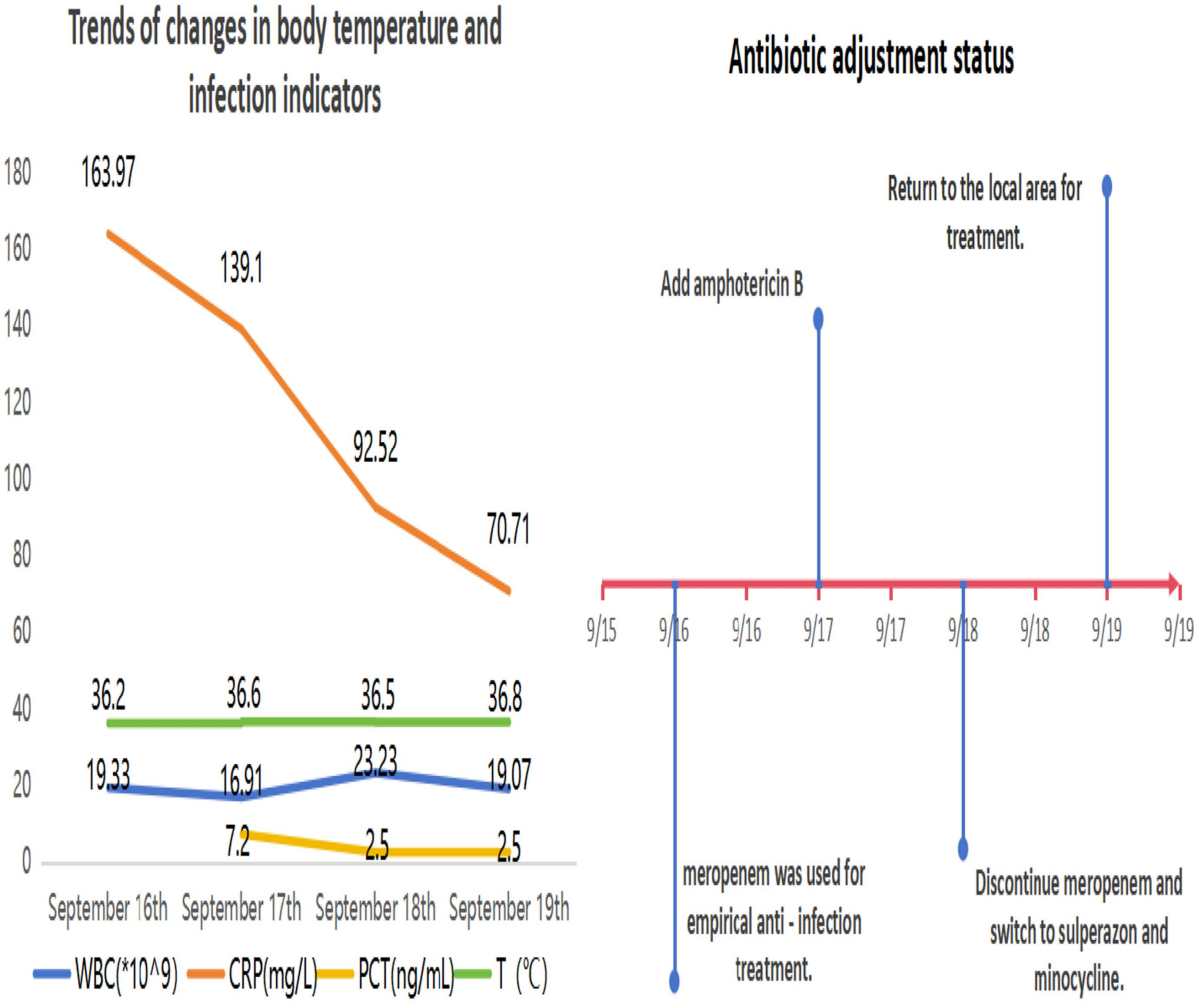
Timeline of clinical course, antimicrobial therapy, and trends of key indicators.

**Figure 4:**
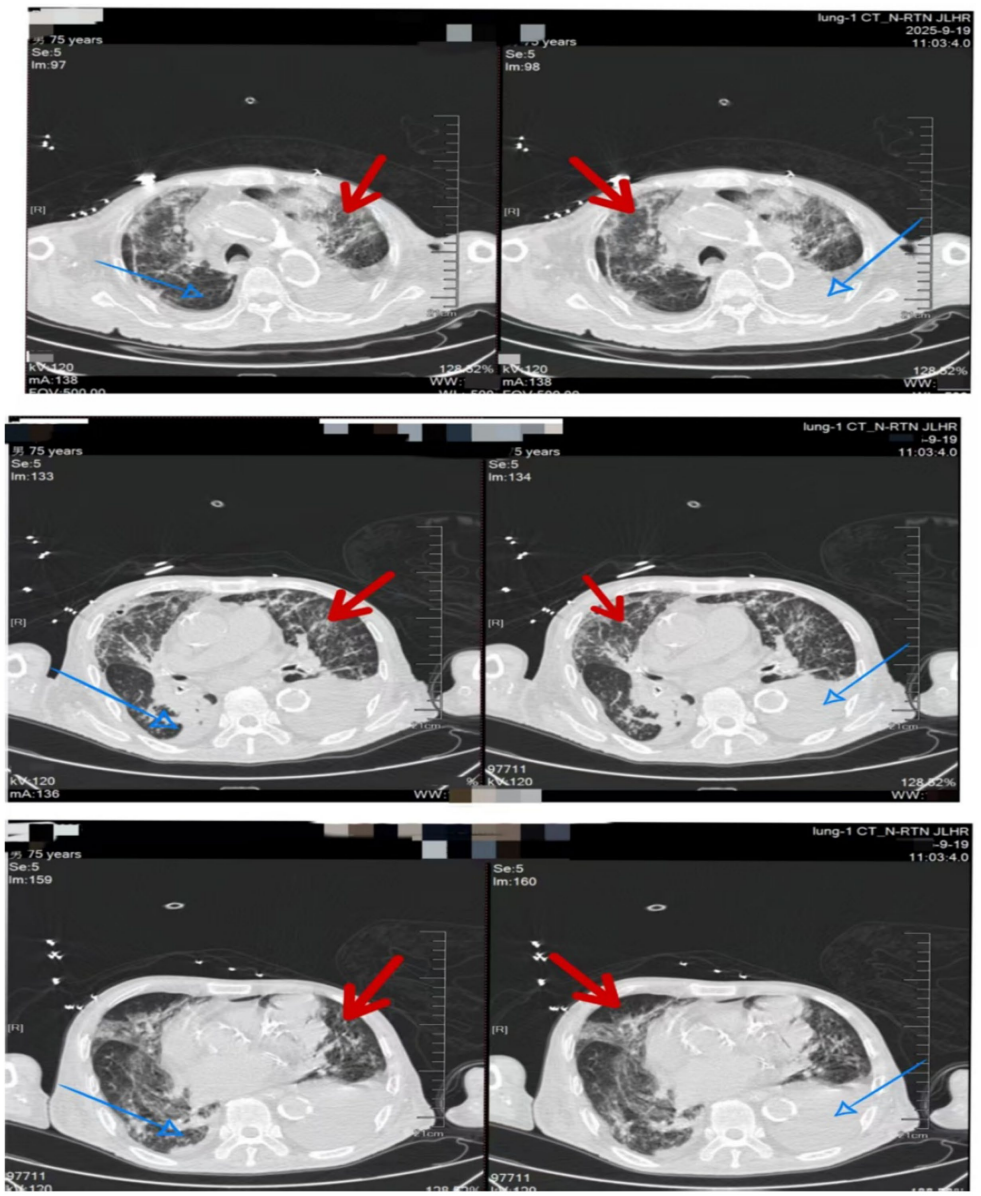
Chest CT image of the patient obtained 3 days after initiation of targeted treatment. Blue arrows indicate expansion of the right lower lobe compared to admission, suggesting improvement of atelectasis. Red arrows demonstrate reduced scattered high-density opacities in the upper lobes of both lungs, indicating gradual resolution of pulmonary inflammation.

Upon admission, given the patient’s impaired consciousness and critical condition, clinical decision-making followed the principle of empirical followed by targeted anti-infective therapy. Sputum and BALF samples were collected for routine bacterial culture and identification. Both qualified sputum and BALF cultures were subsequently identified as *C. gilardii*. However, the clinical microbiology laboratory was unable to perform AST for this isolation. Concurrent targeted pathogen DNA sequencing also detected *S. maltophilia* (dominant species), along with *Candida glabrata* and *Candida albicans*. In the absence of susceptibility data, the initial empirical antimicrobial regimen consisted of meropenem combined with amphotericin B cholesteryl sulfate complex. Adjunctive therapies included continuous renal replacement therapy (CRRT), mucolytic agents, and therapeutic bronchoscopy with lavage. Upon receipt of the microbiological identification, meropenem was discontinued due to the documented intrinsic resistance of both *C. gilardii* and *S. maltophilia* to carbapenems. The regimen was then adjusted to cefoperazone–sulbactam (Sulperazon) combined with minocycline. After this targeted adjustment, the patient’s inflammatory markers showed significant improvement (Figure 3), and follow-up chest CT revealed partial resolution of pulmonary infiltrates (Figure 4).

## Discussion

*Cupriavidus gilardii* has historically been described as a low-pathogenic organism that typically causes opportunistic infections. Traditionally classified as an environmental saprophyte and an opportunistic pathogen of low virulence, it primarily inhabits water, soil, and environments contaminated with heavy metals (11–13). To date, most reported human infections have occurred in hosts with defined immuno-compromising conditions, such as hematologic malignancies, solid organ transplantation, or those receiving immunosuppressive therapy. This established perception has contributed to a lack of clinical vigilance toward this bacterium. However, emerging case reports over the past few years are gradually challenging this conventional understanding. For example, Kobayashi et al. (7) and Zhang et al. (14) have separately reported cases of *C. gilardii* infection in individuals without underlying immunocompromising conditions. These findings strongly suggest that the pathogenic potential of this bacterium may have been substantially underestimated. It appears capable of breaching intact immune barriers under specific circumstances, indicating it is not strictly an opportunistic pathogen. This evolving epidemiological profile necessitates that clinicians and microbiologists, when encountering this organism in the future, consider it a potential pathogen rather than mere contamination or colonization, regardless of the patient’s immune status. Our patient exemplifies a “classic high-risk host” for *C. gilardii* infection. His risk factors can be analyzed across three interconnected dimensions. Firstly, his baseline condition—advanced age, chronic renal insufficiency (on maintenance hemodialysis), bilateral lower limb amputation, and severe hypoalbuminemia—collectively constituted a state of significant acquired immunodeficiency. Chronic renal insufficiency itself is associated with immune dysfunction, and malnutrition further compromises immune responses (15, 16). Secondly, iatrogenic factors in this case provided critical portals for the colonization and invasion of *C. gilardii*. The patient had a long-term subcutaneous tunneled central venous catheter, established after the failure of his arteriovenous fistula. The biofilm that typically forms on the surfaces of such devices (16, 17) could have served as a potential “sanctuary” for environmental bacteria such as *C. gilardii*. Although blood cultures failed to detect the bacterium, the possibility of an occult focus at this site, with subsequent dissemination of the lungs, cannot be excluded. This potential mechanism aligns with documented cases of *C. gilardii* infections involving pacemakers and catheter-related bloodstream infections (7, 11). While the patient had no definitive history of soil exposure, the hospital water environment (e.g., irrigation solutions, faucets) must be considered a potential nosocomial source for acquiring this organism (6). Finally, regarding the acute precipitating factor, aspiration secondary to nasogastric tube feeding caused acute chemical lung injury, severely compromising the integrity of the respiratory mucosa. This created an optimal environment for the proliferation of *C. gilardii*, whether it was already colonizing the respiratory tract or had hematogenous disseminated to the lungs, ultimately leading to the development of severe pneumonia dominated by this pathogen.

Given the patient’s prolonged healthcare exposure and the environmental nature of *C. gilardii*, a nosocomial acquisition warrants serious consideration. The assessment can be framed around three potential reservoirs or portals. First, invasive devices played a likely central role. The patient’s long-term tunneled central venous catheter, necessary for hemodialysis, represents a prime candidate for biofilm formation and subsequent bacterial sanctuary, potentially leading to occult catheter colonization with eventual hematogenous seeding to the lungs—a mechanism documented in prior *C. gilardii* catheter-related bloodstream infections (5, 10). Second, the hospital environment itself is a known reservoir for various waterborne *Cupriavidus* species. Potential sources include contaminated hospital water supplies (tap water, sinks), inadequately disinfected respiratory therapy equipment (e.g., nebulizers, ventilator circuits), or even prepared irrigation/sterile solutions, as suggested by its recovery from similar niches in other reports (4, 16). Finally, healthcare-associated procedures could have introduced the organism. Therapeutic bronchoscopy, performed for lavage, or routine manipulation of the central venous catheter, while performed under aseptic technique, remain potential vectors if any breach in protocol occurred or if a contaminated solution was used. However, it is crucial to acknowledge the limitations of this assessment. Without positive cultures from the catheter tip, environmental samples, or molecular typing to match clinical and environmental isolates, the precise mode and source of acquisition remain speculative. Nonetheless, this case reinforces the importance of stringent infection control practices around water sources and invasive device management, particularly in units caring for highly immunocompromised patients.

Our case must be understood within the context of the growing yet still sparse literature on *C. gilardii* infections, including recent reports in patients with chronic kidney disease, immunocompromised states, and post-COVID-19 convalescence. These prior cases have collectively established key knowledge: the bacterium’s role as an opportunistic pathogen in vulnerable hosts and the recurrent, system-level challenge posed by the absence of standardized AST. However, our report addresses a critical gap illuminated by these earlier studies. While they document the existence of the AST dilemma, they often describe scenarios with relatively discrete immunocompromising conditions, and some even report isolate-specific susceptibility data that, while non-standardized, offered some guidance. The unresolved question was: how does this diagnostic-therapeutic gap manifest and intensify in the face of extreme host complexity and a complete absence of any interpretable laboratory guidance? Our case provides a definitive, and stark, answer. It demonstrates that in a host with profound, multi-system vulnerabilities (end-stage renal disease, severe malnutrition, physical disability), the consequences of the AST void are magnified. The inability to perform conventional AST on our isolate forced a transition from empirical therapy to targeted treatment based solely on historical case reports—a “best-guess” approach devoid of contemporary, isolate-specific evidence. This scenario moves the discussion from acknowledging a general problem to illustrating its concrete, high-stakes impact on real-time decision-making in a critically ill patient. Furthermore, the patient-requested premature cessation of therapy due to insurmountable socioeconomic factors adds a crucial, often-overlooked dimension to the narrative of managing emerging pathogens, highlighting how non-biological barriers can dictate outcomes even when a clinical response is initiated. Thus, the new insight contributed by this case is twofold: it vividly operationalizes the abstract challenge of non-standardized AST in a context of extreme clinical complexity, and underscores the intersection of microbiological uncertainty with broader healthcare delivery challenges.

The initial empirical regimen of meropenem combined with amphotericin B was selected based on the patient’s presentation with severe pneumonia, history of aspiration, and prior broad-spectrum antibiotic exposure, aiming to provide coverage against common nosocomial pathogens, including drug-resistant Gram-negative bacilli and fungi. However, subsequent microbiological findings revealed the limitations of this approach: *C. gilardii* has been frequently reported to exhibit intrinsic resistance to carbapenem antibiotics such as meropenem (12, 17, 18), and the co-isolated *S. maltophilia* is also inherently non-susceptible to this class. This dilemma compelled the treatment team to seek alternative regimens based on limited published evidence (19). According to a report by Karafin et al. involving a pediatric patient with aplastic anemia (9), along with other scattered case reports, *C. gilardii* may retain *in vitro* susceptibility to trimethoprim–sulfamethoxazole, fluoroquinolones, tetracyclines, and certain *β*-lactam/β-lactamase inhibitor combinations (20). This susceptibility partially overlaps with that of *S. maltophilia* in our institution.

The absence of standardized AST data fundamentally complicated management. The empirical use of meropenem created a vulnerable “diagnostic-therapeutic gap,” exposing the patient to an ineffective drug during the critical initial phase. Subsequent therapy adjustment was a “best-guess” strategy based on extrapolation from scarce case reports rather than isolate-specific evidence. This lack of AST data precluded confident de-escalation, blurred the definition of optimal treatment duration, and hampered early detection of potential therapeutic failure, highlighting a systemic challenge in managing emerging, multidrug-resistant pathogens.

In the present case, the isolation of *C. gilardii* from two qualified respiratory specimens, in the context of severe clinical deterioration and no other dominant pathogen, argues for its pathogenic role. However, we acknowledge the limitations. The patient-requested premature transfer resulted in a relatively short observation period for targeted therapy. While initial clinical and radiological improvement was noted, this abbreviated course limited our ability to perform follow-up microbiological studies to definitively exclude a contributory role of other microorganisms detected by molecular methods (e.g., *S. maltophilia*). Furthermore, the inability to perform standardized antimicrobial susceptibility testing remains a central challenge. So, we cannot definitively rule out colonization. Therefore, we have cautiously referred to *C. gilardii* as a probable pathogen. This underscores the diagnostic challenge posed by such rare environmental organisms.

## Conclusion

This case illustrates the severe disease *C. gilardii* can cause in critically ill, immunocompromised hosts and challenges its perception as a low-virulence opportunist. The intrinsic resistance of this pathogen to common empiric antibiotics, coupled with the lack of standardized susceptibility testing, creates a dangerous therapeutic gap. To improve outcomes, there is an urgent need for multidisciplinary awareness, rapid diagnostic techniques, and the development of standardized AST guidelines through multicenter collaborations for such emerging environmental pathogens.

## Data Availability

The original contributions presented in the study are included in the article/supplementary material, further inquiries can be directed to the corresponding author.
